# Receptor-Mediated Gene Delivery

**DOI:** 10.1100/tsw.2002.95

**Published:** 2002-01-24

**Authors:** Tatjana C. Gust, Martin Zenke

**Affiliations:** Max Delbrück Center for Molecular Medicine (MDC), Robert-Rössle Str. 10, D-13125 Berlin, Germany

**Keywords:** gene delivery, gene therapy, receptor-mediated gene transfer, vectors

## Abstract

Receptor-mediated gene delivery capitalises on the presence of specific cell surface molecules for DNA uptake into cells and represents a particularly appealing approach for targeting vector DNA to specific cell types in vivo and in vitro. Various ligand/DNA and antibody/DNA transfer complexes were generated that, following binding to cells, are internalised and reach the endosomal compartment. Vector complexes contain endosomolytic components that ensure vector release from the endosome and translocation of vector DNA into the nucleus where transcription occurs. Thus, receptor-mediated gene delivery encompasses several critical steps that must be considered when designing and applying such vector systems.

## INTRODUCTION

Gene therapy of diseases, such as cancer or hereditary diseases, requires selective gene delivery into the affected cells. Selective gene delivery should ensure transgene expression only in target cells and should be effective, safe, and readily applicable. One method of selective transgene delivery targets cell-surface–bound receptors, since many receptors are expressed in a cell-type–specific manner, such as asialoglycoprotein and mannose receptors, or are highly expressed in malignant cells, e.g., transferrin receptor. When designing an optimal vector system for receptor-mediated gene transfer, several steps that are crucial for efficient transgene delivery and expression have to be considered.

Receptor-targeted gene delivery into cells involves several steps:

1. The vector must specifically bind to cognate receptor.
2. The vector/receptor complex has to be internalised. Thus, only receptors that are internalised upon ligand binding are suitable targets for receptor-mediated gene delivery.
3. Following internalisation of the vector/receptor complex, vector DNA has to be released from the endosomal compartment.
4. The vector DNA has to translocate into the nucleus.

Thus, vector systems frequently include agents that ensure protection of vector DNA from endosomal degradation and effective DNA release into the cytoplasm. Following escape from the endosome, vector DNA translocates into the nucleus and transcription occurs. Nuclear translocation represents a particularly strong barrier for efficient gene delivery, especially in nonproliferating cells where the nuclear membrane stays intact. In this case, translocation of DNA relies on active transport mechanisms that so far are poorly understood. Over the past several years, many receptor-specific ligands, endosomolytic components, and signals for nuclear translocation have been identified and are being further investigated.

Most clinical protocols in gene therapy used today employ viral vector systems, since viral vectors contain highly evolved and specialized components and are therefore very effective in both transgene delivery and expression. However, viral vectors are frequently associated with cytotoxic side effects and can induce antiviral immune responses. For these reasons, synthetic gene delivery systems are becoming increasingly important. This review summarizes the current state of such synthetic vector systems.

## VECTORS FOR RECEPTOR-MEDIATED GENE DELIVERY

For transgene delivery via cell surface receptors, vector DNA has to be condensed and complexed with receptor ligands. Given the negative charge of DNA, polycationic agents are most suitable for this purpose. The polycations protamine, polylysine (pL), and polyethylenimine (PEI) were found to be effective in complexing DNA with receptor ligands[1,2]. Furthermore, PEI possesses endosomolytic activity that facilitates DNA release into the cytoplasm. PEI was shown to lead to endosome swelling and disruption by acting as a “proton sponge”[3,4,5]. Thus, PEI represents the most frequently used agent for DNA condensing in receptor-mediated gene delivery.

To specifically target receptors, several ligands have been employed, such as asialoglycoprotein[6,7,8,9,10,11], transferrin[12,13,14], folate[15], mannose[16,17,18,19,20], and epidermal growth factor[21,22] or their respective analogues (Table 1). Asialoglycoprotein and transferrin receptor (TfR) were the first receptors employed for selective gene delivery[6,7,8,12,13,14]. Asialoglycoprotein receptor is abundantly expressed on hepatocytes and thus is particularly suited for gene therapy of liver diseases, yet only limited applications have been reported so far[9,10,11,23]. Asialoglycoprotein/pL or galactose/pL, or the respective PEI conjugates contain-ing vector DNA, were used to transfect murine or human hepatocyte-derived cell lines *in vitro* or to directly target hepatocytes *in vivo* in mouse models[6,7,23,24,25,26]. Alternatively, vector DNA was packaged into liposomes that were modified with galactose or other asialoglycoprotein-related proteins, and delivered into liver cells and transgene expression was detected[27].

TfR is widely expressed on actively dividing cells and therefore is particularly appealing for gene delivery into highly proliferating cells, such as tumour cells. In addition, after loading with iron-bound transferrin, the transferrin/TfR complex is internalised, and following iron release in the endosome, the apotransferrin/TfR complex is recycled and transported back to the cell surface[1]. Here the TfR can be recharged, thereby allowing the repeated internalisation of ligand molecules. Various DNA binding polycations, liposomes, or streptavidin-DNA conjugates that are bound to iron-loaded transferrin resulted in efficient gene delivery into many cell types[12,13,14,28,29,30]. Additionally, TfR-mediated gene delivery was successful *in vivo* after modification of the complex formulation. Covalent coupling of polyethylene glycol to PEI/DNA transferrin complexes efficiently reduced toxicity of the complex mainly by reducing complement recruitment, aggregation, and nonspecific binding, and this might account for the improved targeting of DNA transfer complexes and allowed systemic application in mice[31]. Furthermore, the application of transferrin-shielded PEI/DNA complexes yielded preferential transgene expression in tumours in mouse tumour models[32]. Finally, TfR-mediated gene delivery was

**TABLE 1**
**Gene Delivery Systems that Target Cell Surface Receptors and Other Cell Surface Molecules**

| Targeted Cell Surface Molecule | Transfection Complex |
|---|---|
| Asialoglycoprotein receptor | Asialoglycoprotein-pL/DNA |
| | Galactose-pL/DNA |
| | Galactose-PEI/DNA |
| | Galactose or asialoglycoprotein modified liposomes + DNA |
| Transferrin receptor | Transferrin liposomes/DNA |
| | Transferrin-pL/DNA |
| | Transferrin-PEI/DNA |
| | PEGylated transferrin-PEI/DNA |
| Mannose receptor | ManpL/DNA |
| | ManPEI/DNA |
| | Mannosylated cationic liposomes/DNA |
| Monoclonal antibodies | Anti-CD3 antibody/PEI/DNA |
| | IgG/pL/DNA |
| | Anti-B lymphoma antibody/pL/DNA |
| Fab fragments | Fab of anti-EGFR/pL/DNA |
| scFv | ErbB2-specific scFv/protamine/cationic lipids/DNA |
| | pIgR-specific scFv/pL/DNA |

employed to generate autologous IL-2 modified melanoma cells in a Phase I clinical trial in patients with metastatic malignant melanoma[33,34,35].

Mannose receptor and mannose-receptor–related receptors are abundantly expressed on antigen-presenting cells, such as macrophages and dendritic cells, and are important for endocytosis and phagocytosis of a variety of antigens exposing mannose and fucose residues. In addition, mannose receptors are recycled and transported back to the cell surface similarly to TfR, thus allowing repeated internalisation of new ligand molecules[36]. For this reason, mannose receptors are well suited for targeted delivery of DNA into cells by employing synthetic mannose (Man) polycation conjugates and liposomes, and using a strategy that was successfully employed before for TfR. Accordingly, ManpL and ManPEI/DNA transfer complexes and mannosylated cationic liposomes were generated and applied for DNA delivery into macrophages and dendritic cells[16,17,18,19,20]. Furthermore, such gene-modified dendritic cells were effective in antigen presentation and elicited potent antigen-specific T cell responses[18,19]. The incorporation of adenovirus particles in DNA transfer complexes further enhances transgene expression.

## TARGETING VECTORS FOR CELL SURFACE MOLECULES

Yet another approach for targeting cell-type–specific surface molecules is the use of monoclonal antibodies, antibody fragments that bind the antigen (Fab), and single-chain antibody fragments (scFv) consisting of the variable domains of light and heavy chain[35,36,37,38,39,40,41,42,43] (Table 4). Monoclonal antibodies that target surface markers on human peripheral blood mononuclear cells (PBMC), such as CD3, and Fc receptor on macrophages were investigated. Anti-CD3 antibody coupled to PEI efficiently transduced T lymphocytes and PHA-stimulated PBMC[38,43]. Similarly, IgG was efficient for Fc receptor–mediated gene delivery into alveolar macrophages[37]. Furthermore, murine and human B lymphoma cell lines were effectively transduced with reporter DNA, i.e., beta-galactosidase and luciferase DNA, by using B-lymphoma–specific monoclonal antibodies conjugated to polylysine[40]. Fab fragments of the antihuman epidermal growth factor receptor (EGFR)

antibody conjugated to pL efficiently bound DNA and successfully targeted EGFR-hyperproducing tumour cells[44,45].

scFv have several advantages over full-size antibodies. Because of their small size, they extravasate rapidly from blood and penetrate faster and deeper into tissues. Since they lack the constant region of the antibody molecule, they are not bound by Fc receptors and retained in tissues such as the liver and kidney. Thus possible side effects are reduced[46]. Different scFv were produced and employed in receptor-mediated gene delivery. For example, ErbB2-positive human breast cancer cells were selectively transduced by a complex containing an ErbB2-specific scFv, protamine, cationic lipids, and vector DNA[42]. Additionally, human polymeric immunoglobulin receptor (pIgR) expressing cells of the airway epithelium were readily transfected by pL DNA complexes containing an scFv specific for the pIgR[41].

Finally, in some vector systems viral components were included to take advantage of the highly efficient transduction potential of viruses. For example, in adenovirus/PEI/DNA (Ad/PEI/DNA) transfer complexes, plasmid DNA is bound to the outside of adenovirus particles, and such complexes deliver DNA into cells via the adenovirus internalisation route[19,47,48]. In this gene delivery system PEI serves both as a DNA-condensing agent and linker for binding the PEI/DNA complex to virus particles through charged interactions with negative domains on the viral hexon. Genetically and UV/psoralen-inactivated adenovirus particles are employed that do not show viral gene expression and thus minimize potential antiviral immune responses[47].

In summary, a large number of synthetic and semisynthetic vector systems that rely on targeted gene delivery are currently being developed. These vector systems target a variety of cell surface molecules, such as receptors or other cell surface determinants. Additionally, while a receptor-specific targeting is readily achieved in several *in vitro* systems, this appears to be more difficult *in vivo*. This can be due to several causes, such as inactivation and unspecific binding of transfer complexes, limited accessibility of target cells, and immune responses to vectors. Thus, further developments in vector formulations are clearly required to translate such gene delivery systems into routine use in medical therapy.

## ACKNOWLEDGEMENTS

Part of this work was funded by the Deutsche Forschungsgemeinschaft (DFG; SFB 506) to M.Z. and a grant of the Edward Jenner Institute for Vaccine Research (EJIVR) to T.C.G. and M.Z.
